# Impact of Agroforestry Practices on Soil Microbial Diversity and Nutrient Cycling in Atlantic Rainforest Cocoa Systems

**DOI:** 10.3390/ijms252111345

**Published:** 2024-10-22

**Authors:** Sayure Mariana Raad Nahon, Felipe Costa Trindade, Caio Augusto Yoshiura, Gabriel Caixeta Martins, Isa Rebecca Chagas da Costa, Paulo Henrique de Oliveira Costa, Héctor Herrera, Diego Balestrin, Tiago de Oliveira Godinho, Bia Makiyama Marchiori, Rafael Borges da Silva Valadares

**Affiliations:** 1 Programa de Pós-Graduação em Biotecnologia Aplicada à Agropecuária, Universidade Federal Rural da Amazônia (UFRA), Belém 66077-830, PA, Brazil; 2 Instituto Tecnológico Vale (ITV), Rua Boaventura da Silva, 955, Belém 66050-090, PA, Brazil; 3 Departamento de Ciencias Forestales, Facultad de Ciencias Agropecuarias y Medioambiente, Universidad de La Frontera, Temuco 4811230, Chile; 4 Center for Biodiversity and Ecological Sustainability, Facultad de Ciencias Agropecuarias y Medioambiente, Universidad de La Frontera, Temuco 4811230, Chile; 5 Reserva Natural Vale, Rodovia BR 101, km 122 s/n Zona Rural, Linhares 29900-111, ES, Brazil

**Keywords:** metabarcoding, metagenomics, proteomics, soil microorganisms

## Abstract

Microorganisms are critical indicators of soil quality due to their essential role in maintaining ecosystem services. However, anthropogenic activities can disrupt the vital metabolic functions of these microorganisms. Considering that soil biology is often underestimated and traditional assessment methods do not capture its complexity, molecular methods can be used to assess soil health more effectively. This study aimed to identify the changes in soil microbial diversity and activity under different cocoa agroforestry systems, specially focusing on taxa and functions associated to carbon and nitrogen cycling. Soils from three different cocoa agroforestry systems, including a newly established agroforestry with green fertilization (GF), rubber (*Hevea brasiliensis*)–cocoa intercropping (RC), and cocoa plantations under Cabruca (cultivated under the shave of native forest) (CAB) were analyzed and compared using metagenomic and metaproteomic approaches. Samples from surrounding native forest and pasture were used in the comparison, representing natural and anthropomorphic ecosystems. Metagenomic analysis revealed a significant increase in Proteobacteria and Basidiomycota and the genes associated with dissimilatory nitrate reduction in the RC and CAB areas. The green fertilization area showed increased nitrogen cycling activity, demonstrating the success of the practice. In addition, metaproteomic analyses detected enzymes such as dehydrogenases in RC and native forest soils, indicating higher metabolic activity in these soils. These findings underscore the importance of soil management strategies to enhance soil productivity, diversity, and overall soil health. Molecular tools are useful to demonstrate how changes in agricultural practices directly influence the microbial community, affecting soil health.

## 1. Introduction

Brazil is widely recognized as one of the world’s most important biodiversity hotspots, with the Amazon, Cerrado, Atlantic Forest, and Pantanal biomes being particularly rich in biodiversity and playing a critical role in soil carbon sequestration [1,2,3]. However, these ecosystems are under significant threat from a variety of disturbances, including deforestation, mining, cattle ranching, and conversion to agricultural or forestry systems, which negatively impact natural forest regeneration [4,5]. Given the current rate of biodiversity loss, it is critical to develop strategies to conserve native biodiversity at multiple scales.

Agroforestry systems offer a promising approach by integrating agricultural and forestry crops to promote sustainable land management while supporting biodiversity conservation. A notable example is the cultivation of *Theobroma cacao* L. (cocoa), which is of great economic importance in Brazil. The state of Espírito Santo, particularly the municipality of Linhares, is a major cocoa producer, accounting for 70.7% of the state’s total production [6]. Cocoa-based agroforestry systems have shown numerous benefits, including increased yields, improved soil micronutrient levels, and enhanced carbon storage compared to monoculture systems [7,8]. In addition to supporting economic development, these systems provide important ecological services, such as increasing soil carbon and nitrogen storage and enhancing microbial functional diversity [9,10,11,12,13]. Such benefits reduce the need for chemical inputs and contribute to sustainable land management. However, further research is needed to optimize productivity, particularly by exploring plant–soil interactions that can mitigate biodiversity loss, reduce production costs, and improve soil quality [14].

Soil quality refers to the ability of soil to support biological productivity, maintain environmental quality, and promote plant and animal health, with its chemical, physical, and biological components serving as key indicators [15]. Microbial diversity, which is influenced by factors such as plant species and land use, is a critical indicator of soil biological quality because of its role in biogeochemical cycles such as carbon and nitrogen [16,17,18,19]. While traditional biochemical assays or direct measurements of biological activity provide only a limited snapshot of the complex and dynamic interactions within soils, molecular tools offer deeper insights into processes such as nutrient cycling and symbiosis [20]. By integrating molecular techniques, we can achieve a more comprehensive and nuanced understanding of soil ecosystems, ultimately leading to more effective soil health management strategies [21]. Although studies using metabarcoding have examined microbial composition, approaches such as metaproteomics and metagenomics are essential for understanding the metabolic processes and microbial diversity in response to land-use changes and agroecosystems [22,23,24].

Few studies have analyzed the impact of cocoa agroforestry systems on the community structure of soil microorganisms in the Brazilian Atlantic Rainforest, and the identification of active microorganisms remains a largely unexplored area. To better understand the role of these microbial communities in nutrient cycling, it is crucial to identify shifts in their composition. A practical approach is to analyze the genes and proteins associated with soil processes, which reveals the functional capacity of microbial communities [25]. In this research, we hypothesized that cocoa agroforestry systems promote greater diversity and improved carbon and nitrogen cycling, thereby enhancing soil quality. Therefore, the aim of this study was to reveal the impact of cocoa agroforestry systems on the soil microbial structure and functional profiles of taxa involved in carbon and nitrogen cycling in the Southeast Atlantic Rainforests of Brazil. To the best of our knowledge, this is the first study to investigate the impact of different cocoa agroforestry systems on the structure of active microbial communities using a combination of high-throughput sequencing methods.

## 2. Results

### 2.1. Chemical and Physical Soil Characterization

The pasture (PAS) and agroforestry system with green fertilization (GF) had lower soil organic matter (SOM) and nitrogen (N) accumulations compared to the Cabruca agroforestry system (CAB), rubber tree (*Hevea brasiliensis*)–cocoa intercropping (RC), and native forest (NF) areas (Figure S1). The median ± MAD values for SOM were 1.06 ± 0.37, 1.44 ± 0.62, 2.47 ± 1.33, 2.53 ± 0.89, and 2.15 ± 0.99%, and for N were 0.08 ± 0.02, 0.08 ± 0.05, 0.23 ± 0.08, 0.21 ± 0.08, and 0.16 ± 0.08% for PAS, GF, CAB, RC, and NF, respectively. Soil pH values ranged from 4.0 to 7.2, with the lowest median values found in the NF (4.3 ± 0.15) and PAS (4.8 ± 0.15) plots. These two areas also had the highest values for aluminum saturation (NF = 61 ± 26% and PAS = 66 ± 25%) and the lowest values for base saturation (NF = 13 ± 7% and PAS = 18 ± 11%). The CAB area showed the highest values of phosphorus (P = 5.3 ± 0.9 mg/dm^3^), manganese (Mn = 151 ± 37 mg/dm^3^), and copper (Cu = 1.6 ± 0.7 mg/dm^3^) compared to the other areas (Figure S1). For example, the PAS area presented 1.9 ± 0.6 mg/dm^3^, 0.7 ± 0.6 mg/dm^3^, and 0.1 ± 0.0 mg/dm^3^ of P, Mn, and Cu, respectively. In general, the cation exchange capacity (CEC) values followed a similar trend as the SOM values. The soils in the Cabruca agroforestry system and rubber–cocoa intercropping areas have a clayey texture, while the other areas had a medium texture.

### 2.2. Total Bacterial and Fungal Diversity Identified by Metabarcoding

**Figure 1:**
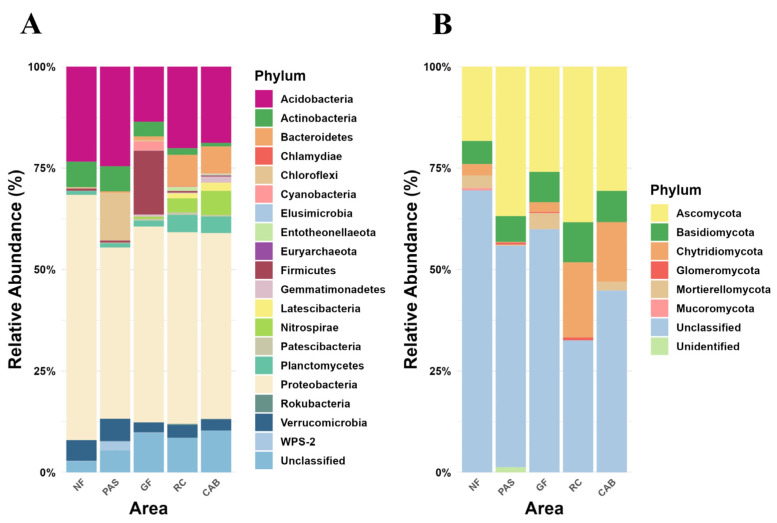
Relative abundance of operational taxonomic units (OTUs) at the phylum level for bacterial (**A**) and fungal (**B**) communities, identified through metabarcoding, in soil samples from native forest (NF), grassland (PAS), green fertilization (GF), rubber tree–cocoa (RC), and Cabruca (CAB) systems.

To detect changes in the community structure of soil microorganisms in response to different cocoa agroforestry systems, fungal and bacterial metabarcoding analyses were conducted. The metabarcoding analysis showed a total of 2,664,675 reads in the 25 16S libraries, which after quality filtering resulted in 1,578,053 sequences for analysis. Overall, there were a total of 114,068 bacterial sequences distributed across 192 distinct operational taxonomic units (OTUs). The Shannon–Wiener diversity index and Pielou Evenness were lower in the native forest, with a Shannon–Wiener index of 4.89 for bacteria and 3.26 for fungi, as well as Pielou Evenness values of 0.76 for bacteria and 0.67 for fungi. In the agroforestry system with green fertilization, the Shannon–Wiener index was 5.14 for bacteria and 3.38 for fungi, with Pielou Evenness values of 0.80 for bacteria and 0.70 for fungi (Figure S2). Proteobacteria and Acidobacteria were the most abundant phyla, accounting for 51% and 22% of the relative abundance, respectively (Figure 1A). At the genus level, uncultured, Candidatus Solibacter and *Acidibacter* were the most abundant, accounting for 52%, 5%, and 4% of the assigned sequences, respectively (Table S1). A higher number of bacterial genera were detected in the agroforestry system with green fertilization (33 genera), rubber–cocoa intercropping (37 genera), and the Cabruca agroforestry system (38 genera) areas (Table S1).

For the 25 ITS libraries, a total of 148,977 reads were obtained, which, after quality filtering, yielded 89,749 sequences for analysis. In total, there were 74,234 fungal sequences distributed among 1008 distinct OTUs. No significant differences were found between the areas regarding fungal diversity as measured by the Shannon–Wiener index (native forest: 0.53; pasture: 0.60; agroforestry system with green fertilization: 1.13; rubber–cocoa intercropping: 1.45; and Cabruca agroforestry system: 1.12). Similarly, for the Pielou Evenness index, the values were as follows: native forest: 0.36; pasture: 0.39; agroforestry system green fertilization: 0.57; rubber–cocoa intercropping: 0.66; and Cabruca agroforestry system: 0.59 (Figure S2). At the phylum level, unclassified, Ascomycota and Chytridiomycota were the most abundant, accounting for 41%, 32%, and 9% of relative abundance, respectively (Figure 1B). Similarly, from the 34 detected genera, unidentified, *Saccharomyces* and *Mortierella* were the most abundant, accounting for 69%, 18%, and 6%, respectively (Table S2). *Mortierella* was significantly abundant in the rubber–cocoa intercropping and Cabruca agroforestry systems.

### 2.3. Active Microbial Diversity Identified by Shotgun Metagenomics

**Figure 2:**
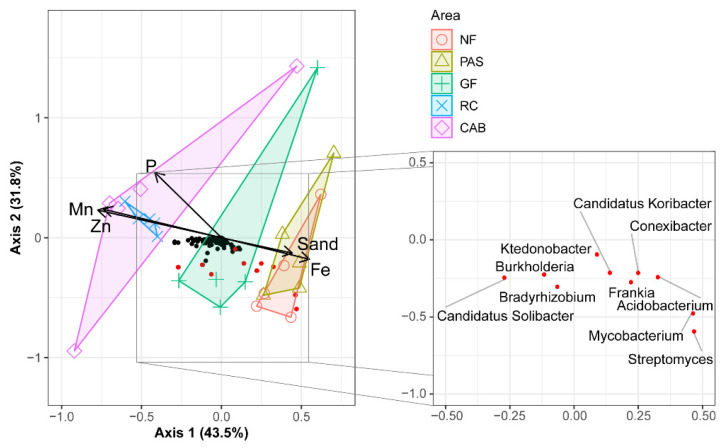
Redundancy analysis (RDA) illustrating the relationships between microbial communities and soil physicochemical parameters, including phosphorus (P), manganese (Mn), zinc (Zn), and iron (Fe) in the native forest (NF), pasture (PAS), green fertilization (GF), rubber tree–cocoa (RC), and Cabruca (CAB) systems. The red points represent the genera that show significant differences between the samples.

The active microbiota involved in carbon and nitrogen cycling across the different soils in cocoa agroforestry systems were identified using shotgun metagenomics. A total of 25 metagenomes were obtained from the different cocoa agroforestry systems and the land uses analyzed in this study. In total, there were 212,455,526 raw reads, which were reduced to 116,564,438 clean reads and 24,653,303 contigs after the quality filtering. A total of 5684 KEGG Orthology identifiers (KOs) were obtained from all samples. The Shannon–Wiener diversity indices and Pielou Evenness in the active taxa showed higher values for bacteria and fungi in the rubber–cocoa intercropping areas. For the rubber–cocoa intercropping, the Shannon–Wiener values were 5.39 for bacteria and 3.51 for fungi, while the Pielou Evenness indices were 0.84 for bacteria and 0.76 for fungi. In the Cabruca agroforestry system, the Shannon–Wiener values were 5.41 for bacteria and 3.52 for fungi, with Pielou Evenness indices of 0.84 for bacteria and 0.78 for fungi (Figure S3). PERMANOVA results for bacteria (0.46) and fungi (0.83) showed that all microbial diversity was affected by sample origin, explaining 41% and 42% of the variance for bacteria and fungi, respectively (*p* < 0.001).The RDA analysis also showed that the sampling site affected the diversity of active microbial communities (Axis 1—43.5% and Axis 2—31.8% of explained variation) (Figure 2). In addition, the samples from the Cabruca agroforestry system, rubber–cocoa intercropping, and green fertilization areas clearly differentiated from those of native forest and pasture, where the variables sand, Fe, P, Mn, and Zn were the factors that significantly shaped the microbial diversity in all the samples (Figure 2).

**Figure 3:**
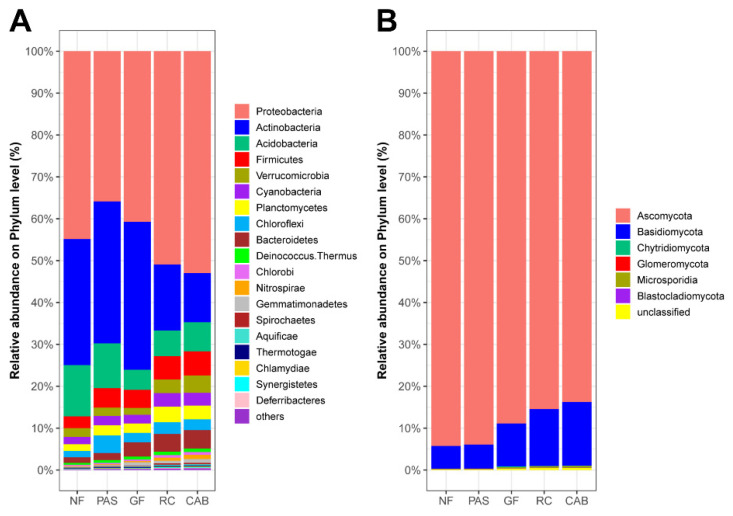
Relative abundance of active taxa involved in carbon and nitrogen cycling at the phylum level for bacterial (**A**) and fungal (**B**) communities, identified through shotgun metagenomics, in soil samples from native forest (NF), grassland (PAS), green fertilization (GF), rubber tree–cocoa (RC), and Cabruca (CAB) systems.

The analysis of the taxa involved in carbon and nitrogen cycling revealed differences in the relative abundance of active taxa in the different soils. Proteobacteria was one of the most abundant taxa with values of 44.87% in the native forest, 35.85% in the pasture, 40.71% in the agroforestry system with green fertilization, 50.93% in the rubber–cocoa intercropping area, and 52.98% in the Cabruca agroforestry system. Similarly, Actinobacteria showed high relative abundances with values of 30.09% in the native forest, 33.90% in the pasture, 35.36% in the agroforestry system with green fertilization, 17.74% in the rubber–cocoa intercropping, and 11.73% in the Cabruca agroforestry system. Ascomycota and Basidiomycota were the most active fungal taxa, with Ascomycota showing a relative abundance of 94.27% in the native forest, 93.95% in the pasture, 88.89% in the agroforestry system with green fertilization, 85.46% in the rubber–cocoa intercropping, and 83.78% in the Cabruca agroforestry system; Basidiomycota accounted for 5.43% in the native forest, 5.73% in the pasture, 10.23% in the agroforestry system with green fertilization, 13.60% in the rubber–cocoa intercropping area, and 15.21% in the Cabruca agroforestry system (Figure 1 and Figure 3). Differential analysis of bacteria revealed a greater abundance of Alphaproteobacteria and Acidobacteria in the native forest (Figure S4). The pasture was significantly enriched with Actinobacteria and Ktedonobacteria, whereas green fertilization showed enrichment of Actinobacteria (Figure S4). In the rubber–cocoa intercropping area, the dominant classes were Deltaproteobacteria, Betaproteobacteria, Gammaproteobacteria, and Planctomycetacia (Figure S4). Finally, in the Cabruca agroforestry system, the most predominant classes were Deltaproteobacteria, Betaproteobacteria, Gammaproteobacteria, and Verrucomicrobiae, showing similarity with the rubber–cocoa intercropping (Figure S4). The differential analysis of fungal taxa (Figure S5) showed similarity between the rubber–cocoa intercropping and Cabruca agroforestry systems, as they presented the fungal classes Saccharomycetes, Agaricomycetes, and Schizosaccharomycetes as preferential (Figure S5). However, in addition to these, the Cabruca agroforestry system exhibits Tremellomycetes and Ustilaginomycetes (Figure S5). The native forest had the highest average difference in the proportion of the fungal classes Eurotiomycetes, Dothideomycetes, and Leotiomycetes, belonging to the phylum Ascomycota. These same classes were also abundant in the pasture area (Figure S5). The class Sordarlomycetes, also belonging to the phylum Ascomycota, showed higher abundance in the agroforestry system with green fertilization (Figure S5).

### 2.4. Genes Involved in C and N Cycling

**Figure 4:**
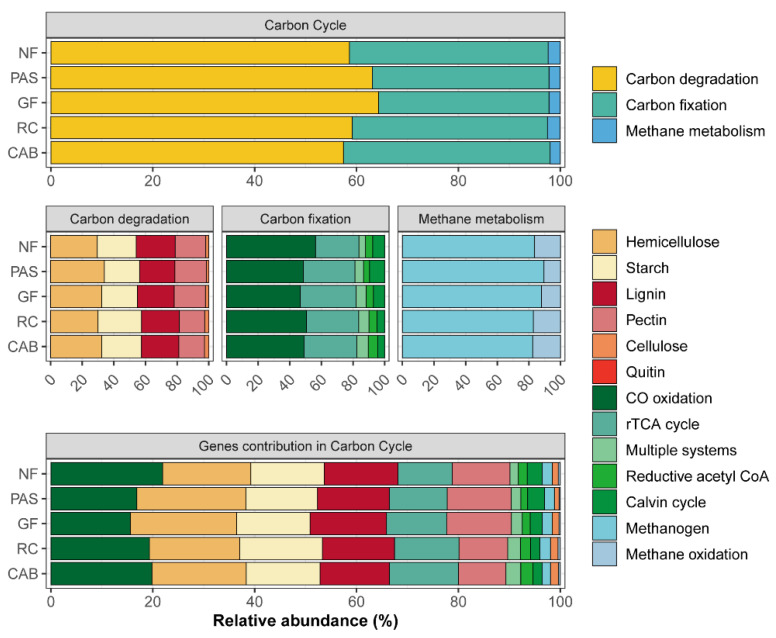
Relative abundance of carbon cycle genes across different land uses, including native forest (NF), pasture (PAS), green fertilization (GF), rubber tree–cocoa (RC), and Cabruca (CAB) systems.

**Figure 5:**
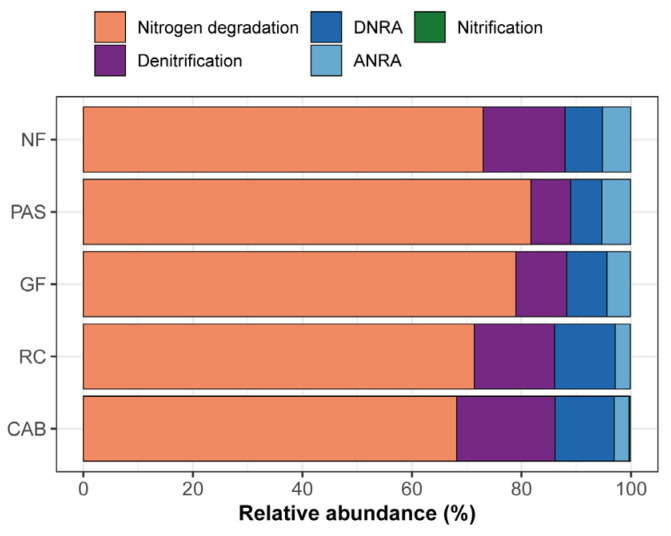
Relative abundance of nitrogen cycle genes across different land uses, including native forest (NF), pasture (PAS), green fertilization (GF), rubber tree–cocoa (RC), and Cabruca (CAB) systems. Nitrogen cycling pathways include DNRA (dissimilatory nitrate reduction to ammonium) and ANRA (assimilatory nitrate reduction to ammonium).

A total of 33 and 13 genes involved in C and N cycling were retrieved from the 25 metagenomes analyzed, respectively. Regarding the functional genes involved in C cycling, they were mainly classified in methane metabolism, carbon fixation, and carbon degradation (Figure 4). Within the functional groups, genes associated with CO oxidation exhibited greater relative abundance: NF: 21.94%, PAS: 16.87%, GF: 15.61%, RC: 19.34%, and CAB: 19.86%. Similarly, genes related to hemicellulose were found in the following abundances: NF: 17.31%, PAS: 21.42%, GF: 20.87%, RC: 17.74%, and CAB: 18.50%. For starch, the relative abundances were as follows: NF: 14.44%, PAS: 14.04%, GF: 14.41%, RC: 16.23%, and CAB: 14.48%. Regarding lignin, the values were as follows: NF: 14.45%, PAS: 14.12%, GF: 14.99%, RC: 14.14%, and CAB: 13.63%. Lastly, carbon fixation genes showed the following abundances: NF: 38.95%, PAS: 34.75%, GF: 33.49%, RC: 38.82%, and CAB: 40.54%. Overall, there were minimal variations among the observed treatments (Figure 4). Also identified were other genes involved in carbon fixation, especially tktA in the native forest, rubber–cocoa intercropping, and Cabruca agroforestry systems. In the pasture area, there was a higher abundance of genes related to carbon degradation compared to the other areas (Figure S6). The genes involved in the N cycle were mainly related to nitrogen degradation and denitrification (Figure 5), with higher relative abundances of genes related to denitrification in the native forest, rubber–cocoa intercropping, and Cabruca agroforestry systems. The metagenome revealed a high abundance of key genes in the N cycle, such as UreC (ammonification) in the native forest, pasture, Cabruca agroforestry system, and rubber–cocoa intercropping areas, being expressed at values of 0.21 for the native forest and pasture and 0.24 for the Cabruca agroforestry system and rubber–cocoa intercropping areas, and the abundance of glutamate synthase in the agroforestry system with green fertilization, with a value of 0.55 (Figure S6).

### 2.5. Active Soil Enzymes

**Figure 6:**
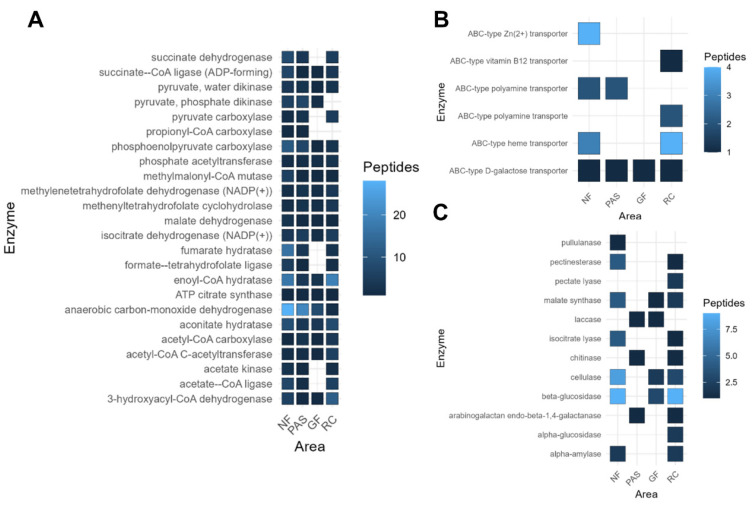
Abundance of soil enzymes associated with key biological processes in different land uses, including rubber–cocoa intercropping (RC), native forest (NF), green fertilization (GF), and pasture (PAS). (**A**) Enzymes responsible for carbon fixation, (**B**) enzymes responsible for nitrogen fixation, and (**C**) enzymes involved in the mineralization of soil organic matter.

Metaproteomic analysis was conducted to identify active soil enzymes involved in carbon and nutrient cycling in the different treatments. Metaproteomic analysis identified 24 enzymes associated with carbon fixation, predominantly found in native forest areas, and 6 enzymes with dehydrogenase activity. In addition, a low abundance of carbon-fixing enzymes was observed in the agroforestry system with green fertilization and rubber–cocoa intercropping areas (Figure 6A). Of the 12 enzymes related to organic matter mineralization, 10 were found in the rubber–cocoa intercropping area. Notably, β-glucosidase was identified exclusively in the green fertilization, rubber tree, and native forest areas, with the highest peptide abundances in the latter two areas (Figure 6C). A greater abundance and similarity of ABC transporters was observed in the rubber–cocoa intercropping and native forest areas compared to the other areas (Figure 6B). These transporters are capable of transporting various substrates across cellular membranes by hydrolysis of adenosine triphosphate (ATP).

## 3. Discussion

### 3.1. Soil Chemical Composition

Soil chemical analyses showed different results in the different study areas. The acidity (average pH) of the soils in the Cabruca agroforestry system and the rubber–cocoa intercropping system can be classified as weakly acidic. In contrast, the native forest and pasture have high acidity (pH < 5.0), while the agroforestry system with green fertilization falls in the range of medium acidity (pH 5.0–5.9), according to the classification of Prezotti, et al. [26]. These pH values also influenced the aluminum saturation (m), with the Cabruca agroforestry system, the rubber–cocoa intercropping area, and the agroforestry system with green fertilization showing low saturation values (<20%), whereas the native forest and the pasture showed high values (>40%). The pH values observed in the pasture are similar to those reported by da Rocha Junior, et al. [27]. In the native forest, soil acidification may be related to organic matter, which releases H+ ions during mineralization, increasing acidity. This process contributes to the typical acidity of tropical soils, which tend to be acidic due to intense leaching of base cations such as calcium (Ca), magnesium (Mg), and potassium (K) [28,29,30].

The highest levels of organic matter and nitrogen were observed in the native forest, rubber–cocoa intercropping, and Cabruca agroforestry systems. Forest-based cocoa agroecosystems typically produce a continuous flow of organic matter into the soil [31]. Monroe, et al. [32] found that leaf deposition in different cocoa agroforestry systems resulted in a thick litter layer, which significantly increased soil organic matter. In addition to the natural senescence of cocoa leaves, pruning practices and the deposition of prunings in the field also contribute to soil organic matter. Thomazini, et al. [33] quantified the effects of different management practices in agroforestry systems and reported similarly high nitrogen levels in forested areas. Furthermore, Schneidewind, et al. [31] demonstrated that cocoa agroforestry systems produce nitrogen-rich litter, highlighting their capacity to store this nutrient in the soil.

### 3.2. Changes in Community Structure of Soil Microorganisms

The results of the effect of agroforestry systems on microbial diversity, obtained through metabarcoding, showed changes in the relative abundance of microorganisms, with Proteobacteria and Ascomycota being the most abundant phyla. This finding is consistent with previous studies highlighting the important role of these groups in soil ecosystems [34,35]. Changes in the structure of soil microbial communities under different cocoa agroforestry systems have been reported previously, supporting the results of this study [36,37]. The conversion of native forest to pasture in the Atlantic rainforest has shown a particular enrichment of microbial taxa such as Firmicutes [38]. Consistent with the present study, Alphaproteobacteria were found to be highly abundant in forest soils [39]. This class includes several important genera, such as *Bradyrhizobium* and *Sphingobium*, which play key roles in nitrogen cycling and the degradation of lignin by-products, respectively [40,41]. In addition, Zhou, et al. [42] observed an increased abundance of Alphaproteobacteria with high nitrogen additions to the soil. Studies have shown that members of the Acidobacteria phylum prefer acidic conditions for growth [43], which probably explains their presence in native forest soils. They are essential for lignin degradation, a crucial process in regulating the soil carbon cycle [44,45]. Other members of Proteobacteria are involved in nitrogen fixation and often form symbiotic relationships with plants [42,46]. This phylum is strongly associated with carbon availability and cycling, showing a positive correlation with the carbon to nitrogen (C/N) ratio [47,48,49,50]. Similarly, Actinobacteria play an important role in nutrient cycling, especially carbon and nitrogen cycling [51,52]. They are essential for the decomposition of plant residues, which enhances carbon sequestration in soils [53], which explains their presence in the agroforestry system with green fertilization.

A similar trend in the relative abundance of fungal taxa was observed by metabarcoding, with many taxa remaining unclassified. However, there was a notable enrichment of Chytridiomycota in the rubber–cocoa intercropping and Cabruca agroforestry systems. Chytridiomycota have been identified as a fungal phylum with strong potential for lignin and cellulose degradation [54], which may be related to the incorporation of plant debris in both systems, and to a lesser extent in the green fertilization agroforestry system and native forest areas (Figure 2). Genera such as *Rhizophlyctis* and *Spizellomyces* are widely recognized for their ability to degrade complex organic matter through various enzymes [55,56]. Similar to the present study, Cerqueira, et al. [57] reported the presence of Ascomycota in forest and pasture areas, probably due to the ability of fungi from this phylum to inhabit diverse environments [58]. The fungal class Tremellomycetes, preferentially associated with Cabruca soils, was also reported by Yu, et al. [59], who found a positive correlation between this class and increased soil organic carbon mineralization. In pasture soils, the abundance of Dothideomycetes was observed, which can attributed to their ability to thrive in challenging substrates [60]. The fungal class Agaricomycetes, found in both Cabruca and rubber–cocoa intercropping soils, contributes to cellulose degradation, thereby increasing nutrient availability and playing a critical role in the carbon cycle [61,62]. Wang and Zhou [63] recently investigated the effect of green fertilization on the fungal composition of the rhizosphere and observed an increase in the abundance of Sordariomycetes with the application of green fertilization. Zhou, et al. [64] and Koechli, et al. [65] also found that cellulose stimulates the growth of this fungal class, which could explain the higher abundance of Sordariomycetes in the green fertilization area.

### 3.3. Microbial Functional Profiles Involved in Carbon and Nitrogen Cycling

The metagenomic results revealed changes in the relative abundance of microbial functional profiles involved in carbon and nitrogen cycling, with a decrease in Actinobacteria and an increase in Proteobacteria and Basidiomycota in the agroforestry systems. Members of Proteobacteria have been identified as key players in soil systems, playing crucial roles in regulating carbon and nutrient cycling, responding to fertilizer inputs, and possessing plant growth promoting traits that can directly enhance plant growth [66,67]. Similarly, Basidiomycota are abundant in forest soils and play a positive role in wood decay and litter decomposition, processes that are enhanced in agroforestry systems [68,69,70].

Nitrogen cycling pathways were particularly enhanced in the agroforestry system with green fertilization (Figure S6), possibly indicating a link between green fertilization and legumes. These results are in line with Hu, et al. [71], who confirmed that legumes stimulate microorganisms involved in nitrogen cycling. Similarly, Lori, et al. [72] showed that agroforestry systems improve soil biological quality. Among the genes identified in the study plots, those related to glutamate synthase showed higher abundance in the agroforestry system with green fertilization (Figure S6). Studies have shown that glutamate synthase is a key enzyme in the GS/GOGAT cycle, which is critical for carbon and nitrogen metabolism [73,74,75]. In addition, UreC, found in the native forest, pasture, Cabruca agroforestry system and rubber–cocoa intercropping areas, encodes urease, an important enzyme derived mainly from soil microorganisms that makes nitrogen available to plants [76,77]. This suggests that the study areas harbor microorganisms that carry this gene and contribute to the nitrogen cycle.

The results also showed that despite changes in overall microbial functional profiles, particularly for bacteria (Figure 3), these changes did not result in the loss of the essential genes involved in carbon and nitrogen cycling (Figure 4 and Figure 5). This may be directly related to the functional redundancy of soil microbial communities, allowing the selective stimulation of microbial taxa capable of maintaining these functions, thus maintaining ecological balance [78,79].

### 3.4. Soil Enzymes Involved in Carbon and Nitrogen Cycling

Metaproteomics facilitated the analysis of soil enzymes, revealing their association with biological processes and positioning them as key indicators of soil quality. Our results showed that enzyme activity varied depending on the management practices applied, highlighting the importance of proper soil management. Enzymes with dehydrogenase activity were detected in rubber–cocoa intercropping and native forest soils, indicating high rates of organic matter decomposition [80]. In addition, recent studies suggest that these enzymes are sensitive indicators of microbial activity, making them valuable for monitoring biological quality and effectively detecting soil changes [81,82,83,84].

A higher abundance of ABC transporters was observed in rubber–cocoa intercropping and native forest soils compared to other areas. ABC transporters are involved in several biological processes, transporting a wide range of substrates across cell membranes by ATP hydrolysis [85]. In soil, certain bacteria possess a significant number of these transporters, which benefit bacterial communities by enhancing nutrient uptake and providing protection against environmental stress [86,87]. Similarly, the cellulase enzyme was more abundant in the native forest, rubber–cocoa intercropping and green fertilization soils, indicating active cellulose decomposition and an increase in organic matter. This finding is in agreement with Florez, et al. [88], who highlighted the influence of vegetation type on cellulose content. Another enzyme closely related to cellulose degradation, beta-glucosidase, was also identified in these areas. This enzyme plays a role in the final stages of cellulose degradation and serves as a reliable indicator of soil quality, providing early indications of changes in organic matter status and turnover [89,90].

Overall, our results show that different agroforestry practices induce changes in the community structure of soil microorganisms, while highlighting the functional redundancy of these communities. This redundancy ensures the conservation of essential genes and enzymes involved in carbon and nutrient cycling. However, this study had significant limitations, including a lack of recent research on soil biology and the effects of agroforestry practices in cocoa systems in the Atlantic rainforest. In addition, the lack of proteomic approaches to assess soil biological quality hindered theoretical development. The soil protein extraction protocol was particularly challenging in the Cabruca agroforestry system, where extraction was unsuccessful due to incompatibilities between the methods used and the soil samples.

## 4. Materials and Methods

### 4.1. Study Area

The study was conducted in areas of the municipality of Linhares, in the state of Espírito Santo, southeastern Brazil. Five different areas (land uses) were evaluated: (i) primary native forest (NF), which is a conserved area with tree growth that is part of the original vegetation; (ii) pasture (PAS), characterized by extensive livestock production with no soil management and numerous areas of exposed soil; (iii) agroforestry system under the implementation of green fertilization (GF), which began in 2020 with the planting of *Gliricidia sepium* using cuttings and mechanical and chemical weeding practices; (iv) rubber tree (*Hevea brasiliensis*)–cocoa intercropping (RC), a well-established and highly productive system combining rubber and cocoa trees, in which the soil is naturally enriched by the leaf litter from the falling rubber tree leaves; and (v) the Cabruca agroforestry system (CAB), consisting of a cocoa plantation established under thinned native forest dominated by “Boleira” (*Joannesia princeps*).

Soil samples were collected from the three different cocoa agroforestry systems: (i) the rubber–cocoa intercropping system (19°03′33″ S, 39°58′55″ W); (ii) the Cabruca agroforestry system (19°24′33″ S, 40°06′16″ W); and (iii) the agroforestry system with green fertilization (19°09′08″ S, 40°04′57″ W). Soil samples from the native forest were collected in the protected area of the Vale Natural Reserve, which is part of a mosaic of protected areas known for its high biodiversity (19°01′36″ S, 40°01′05″ W). The annual rainfall in the area is 1214 mm, with a maximum temperature of 29.9 °C.

In each area, 5 plots of 30 × 30 m were delineated and a total of 3 composite samples were collected within each plot at depths of 0–5 cm, 5–10 cm and 10–20 cm. Soil samples for chemical and physical analyses were placed in plastic bags and sent to the Laboratório Brasileiro de Análises Ambientais e Agrícolas (LABRAS) in Monte Carmelo, MG, Brazil. Samples for biological analysis were collected at a depth of 0–5 cm, placed in a cooler and transported to the Vale Technological Institute—Sustainable Development in Belém, PA, Brazil.

### 4.2. Soil Chemical and Physical Analyses

Soil samples were air dried and sieved through a 2 mm mesh. Analyses were performed according to previous methods [91,92]. Briefly, pH was determined in a water/soil suspension at a ratio of 1:2.5 (*v*/*v*); phosphorus (P) and potassium (K) were extracted with a Mehlich I solution (0.05 mol·L^−1^ HCl and 0.0125 mol·L^−1^ H_2_SO_4_); calcium (Ca), magnesium (Mg), and aluminum (Al) were extracted with a 1 mol·L^−1^ KCl solution; potential acidity (H+Al) was extracted using a SMP buffer solution at pH 7.5; sulfur (S) was extracted with a 0.01 mol·L^−1^ calcium monobasic phosphate solution; boron (B) was extracted with hot water; and copper (Cu), iron (Fe), manganese (Mn), and zinc (Zn) were extracted with a DTPA-TEA solution. The quantifications were performed using an inductively coupled plasma optical emission spectrometer (ICP-OES). In addition, organic matter (OM) was determined by the colorimetric method, and total nitrogen (N) was determined by the Kjeldahl method. The cation exchange capacity (CEC; sum of bases [Ca^+2^ + Mg^+2^ + K^+^ + Na^+^] + H+Al), base saturation (V%; sum of bases/CEC), and aluminum saturation (m%; Al^+3^/sum of bases + Al^+3^) were also calculated. The sand fraction was determined by sieving after chemical dispersion with 0.1 mol·L^−1^ sodium hydroxide and 0.016 mol·L^−1^ sodium hexametaphosphate after stirring for 4 h. The clay fraction was determined after sedimentation using a Bouyoucos hydrometer, and the silt fraction was calculated as the difference between the total mass and the sum of the clay and sand contents.

### 4.3. Fungal and Bacterial Metabarcoding

Total DNA from soil samples was extracted from the top layer (0–5 cm) using the DNeasy PowerSoil Kit (Qiagen, Hilden, Germany) according to the manufacturer’s recommendations. DNA samples were quantified using the Qubit^®^ 3.0 fluorometer (Thermo Fisher Scientific, Waltham, MA, USA) and their quality was verified by 1% agarose gel electrophoresis.

The paired-end metabarcoding amplicon libraries were constructed by PCR amplification of the V3–V4 region of the bacterial 16S rRNA gene and the internal transcribed spacer (ITS) of the fungal 18S rRNA gene. The bacterial primers used were S-D-Bact-0341-b-S-17-N (5′-TCGTCGGCAGCGTCAGATGTGTATAAGAGACAGCCTACGGGNGGCWGCAG-3′) and S-D-Bact-0785-a-A-21-N (5′-GTCTCGTGGGCTCGGAGATGTGTATAAGAGACAGGACTACHVGGGTATCTAATCC-3′), while the fungal primers used were fITS7i (5′-TCGTCGGCAGCGTCAGATGTGTATAAGAGACAGGTGARTCATCGAATCTTTG-3′) and ITS4i (5′-GTCTCGTGGGCTCGGAGATGTGTATAAGAGACAGTCCTCCGCTTATTGATATGC-3′). After amplification, the libraries were verified using the Qubit dsDNA HS Assay (Thermo Fisher Scientific) and the Agilent 2100 Bioanalyzer (Agilent Technologies, Santa Clara, CA, USA) with a DNA 1000 chip. Libraries were purified using the AMPure XP Purification Kit (Beckman Coulter, Brea, CA, USA) and processed using the Nextera XT Kit (Illumina, San Diego, CA, USA). They were then sequenced on the MiSeq-Illumina platform using the MiSeq V3 reagent kit (600 cycles; Illumina) at the Instituto Tecnológico Vale (Belém, PA, Brazil) [93].

### 4.4. Shotgun Metagenomics

Metagenomic libraries were constructed from 50 ng of total DNA and then subjected to a random enzymatic fragmentation reaction using the Illumina DNA Prep Kit (Illumina) according to the manufacturer’s instructions. The fragmented DNA was purified using AMPure XP magnetic beads (Beckman Coulter, Brea, CA, USA) and then subjected to an amplification reaction using primers complementary to the Illumina flow cell adapters. The amplified libraries were re-purified using AM-Pure XP magnetic beads (Beckman Coulter) and quantified using the Qubit^®^ 3.0 fluorometer (Thermo Fisher Scientific). Fragment sizes were verified using a Bioanalyzer 2100 fluorometer (Agilent Technologies, Santa Clara, CA, USA) with a DNA 1000 chip. The libraries were then adjusted to a DNA concentration of 4 nM, pooled, denatured, and diluted to a running concentration of 1.8 pM. Sequencing was performed on the Illumina NextSeq 500/550 platform using the NextSeq 500 v2.5 kit at 300 cycles.

### 4.5. Metaproteomics

The protein extraction from the upper soil layer (0–5 cm) was conducted using an adapted protocol from Qian and Hettich [94]. In 5 g of soil, 10 mL of lysis buffer [100 mM Tris-HCl, pH 8.0, 4% *w*/*v* sodium dodecyl sulfate, and 10 mM Dithiothreitol (DTT)] was added. The samples were placed in a boiling water bath (95 °C) for 10 min in a 1:1 mixture of water and extraction buffer, as described by Ogunseitan [95]. Subsequently, the suspension was sonicated (5 times for 30 s each, at a power of 70 W) and centrifuged at 3200× *g* for 10 min. Ammonium acetate in methanol (0.1 M) was added to the recovered supernatant to precipitate proteins. To clean the samples, the process of adding 1.5 mL of cold acetone (80%) to the microtubes was repeated twice. The tubes were vortexed and centrifuged at 14,000 rpm for 7 min at 4 °C. The supernatant was discarded and 200 µL of 0.1% Rapigest (Waters, Milford, MA, USA) for protein solubilization was added.

The preparation for digestion involved the reduction of proteins with 2 µL of DTT (5 mM) and incubation at 56 °C for 25 min, followed by alkylation with 5.71 µL of iodoacetamide 14 mM for 30 min at room temperature. Residual iodoacetamide was removed by adding 2.09 µL of 5 mM DTT and incubating at room temperature for 15 min. Next, 2.11 µL of 1 mM calcium chloride (CaCl_2_) and 50 µL of 20 ng·μL^−1^ trypsin were added and incubated at 37 °C for 20 h. Then, 1.05 µL of trifluoroacetic acid (TFA; 0.4%) was added and left to incubate at 37 °C for 90 min to stop the enzymatic reaction. Subsequently, the samples were centrifuged at 14,000 rpm at 6 °C for 10 min. The recovered supernatants were transferred to 10 kDa filters and centrifuged for 50 min at 14,000× *g* at room temperature. Finally, the filtered solutions were transferred to vials, dried, and suspended in ammonium formate (20 mM) for effective capture on the first-dimensional column of Ultra-Performance Liquid Chromatography (UPLC). The chromatograph was directly coupled to an ESI-Q-ToF Synapt G2S mass spectrometer (Waters) operating in positive mode and continuous fragmentation (MS E), with collision energy ranging from 5 to 40 eV [96].

### 4.6. Bioinformatics and Data Analysis

For the analysis of the metabarcoding raw data, the paired-end reads were trimmed and filtered using PRINSEQ v0.20.4 and merged using the Paired-End reAd mergeR (PEAR) v0.9.10. The obtained sequences were analyzed using the PIMBA pipeline [97] with the Ribosomal Database Project RDP v11.5 [98] for 16S sequences and the UNITE v8.2 [99] for 18S sequences as reference databases.

For the shotgun metagenomics analysis, the paired-end reads were assembled into contigs using MEGAHIT v1.2.9. Taxonomic classification was performed using Kaiju v1.4.4. The NCBI BLAST non-redundant protein sequences were used as the reference database for taxonomic classification to obtain relative abundance information of taxonomic groups (bacteria, archaea, and fungi). For functional analysis, the paired-end reads were merged using Paired-End reAd mergeR (PEAR) v0.9.10 and submitted to the online tool Ecofun-MAP http://iegst1.rccc.ou.edu:8080/ecofunmap/ (accessed on 15 May 2022) with the following parameters: 10 threads; and workflow: ultra-sensitive.

The proteomic data were analyzed in the Proteinlynx software v.3.0.2 (Waters) for protein identification and quantification using the public database of bacteria, fungi, and plants from Uniprot https://www.uniprot.org/ (accessed on 20 October 2021), as well as data based on the soil metagenomes from the Vale National Reserve. Phylogenetic and functional analyses based on peptides were performed using the Unipept 4.3 software https://unipept.ugent.be/ (accessed on 10 January 2022). In this peptide-centered functional annotation context, biological processes (BPs) related to organic matter mineralization, carbon fixation, and ABC transporters were extracted.

Quantitative data were compared by ANOVA with Tukey’s post-hoc analysis using stats v.4.2.3 and agricolae v.1.3.5 in the R software [100]. The Kruskal–Wallis test followed by the Dunn test (*p* < 0.05) were used to assess the differences of the soil chemical and physical attributes between the land uses. The diversity index calculations were performed using vegan v.2.5.7 and chemodiv v.0.2.0, and then plotted using ggplot2 v3.3.5 in the R environment. Differential analyses were obtained using the software STAMP 2.1.3 [101]. The clustering analysis based on metaproteomics was performed using the Manhattan distance. The sequences obtained in this study have been deposited in the NCBI Sequence Read Archive (http://www.ncbi.nlm.nih.gov/sra/PRJNA1114758) under accession number PRJNA1114758.

## 5. Conclusions

Our approach revealed the effects of different cocoa agroforestry systems on the community structure and functional profiles of soil microorganisms. While several changes in community structure were observed, the functional profiles of microorganisms involved in carbon and nitrogen cycling remained active, highlighting the functional redundancy of soil microorganisms in cocoa agroforestry systems under different management strategies. In particular, the analysis of enzymes related to ABC transporters and organic matter mineralization yielded significant results, especially in rubber–cocoa intercropping, suggesting a positive impact on soil microbial diversity and overall soil health. These results indicate that different cocoa agroforestry systems can improve soil quality by promoting the abundance of active microbial taxa involved in carbon and nutrient cycling. This provides valuable insights into the beneficial role of agroforestry systems in improving soil biological quality, particularly through their effects on functional profiles and active soil enzymes. Future studies should aim to establish links between these functional microbial communities and key ecosystem services such as biomass production, crop yield, and carbon sequestration.

## Data Availability

The sequences obtained in this study have been deposited in the NCBI Sequence Read Archive (http://www.ncbi.nlm.nih.gov/sra/PRJNA1114758) under accession number PRJNA1114758.

## Supplementary Materials

The following supporting information can be downloaded at https://www.mdpi.com/article/10.3390/ijms252111345/s1.
