# Supplementary material for: Impact of Agroforestry Practices on Soil Microbial Diversity and Nutrient Cycling in Atlantic Rainforest Cocoa Systems
Source: Int J Mol Sci. 2024 Oct 22;25(21):11345. doi: 10.3390/ijms252111345 (PMC11545550; doi:10.3390/ijms252111345)
Supplement: Supplementary file 1 [file ijms-25-11345-s001.zip › ijms-3235019-supplementary.pdf]

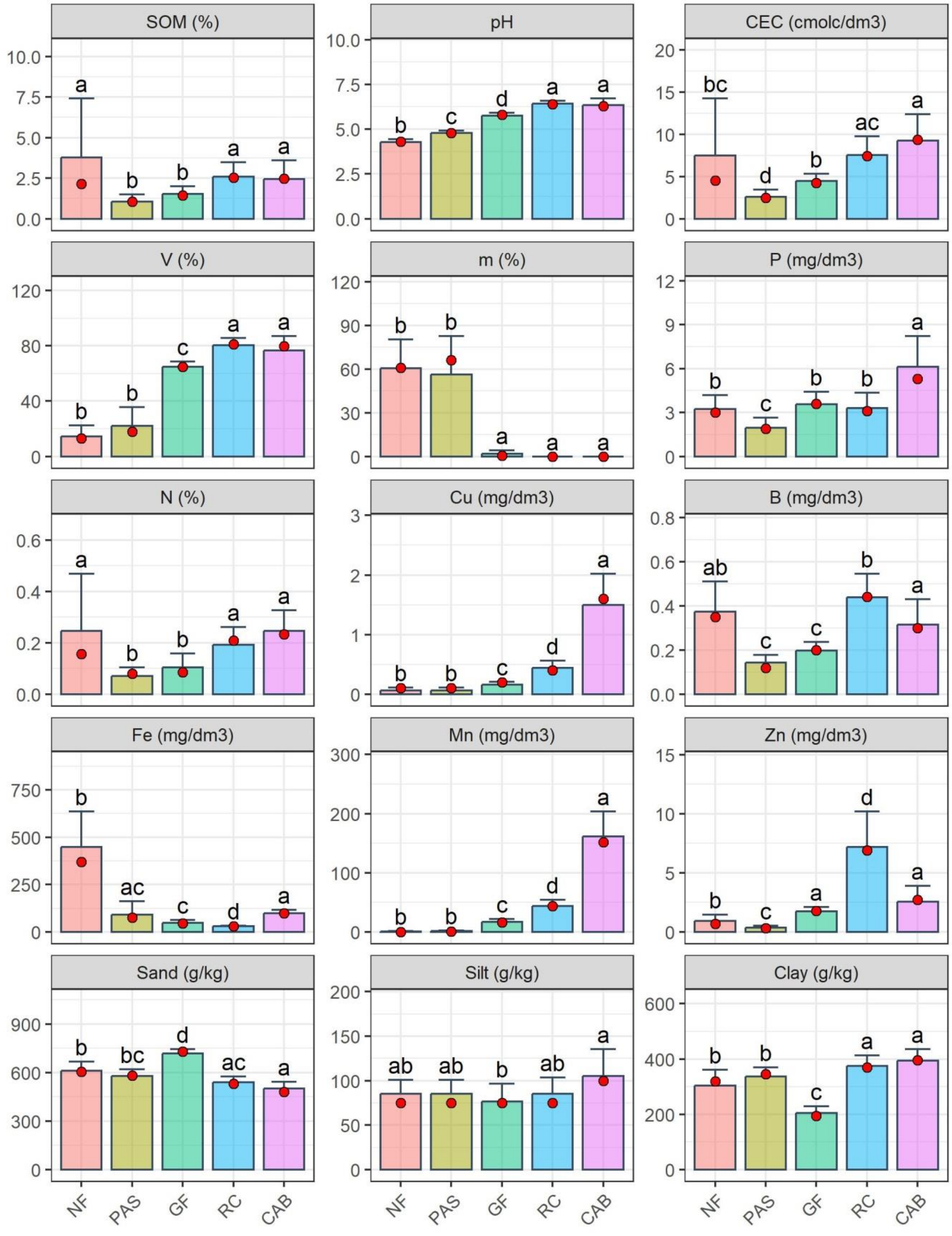

Figure S1. Analysis of physical and chemical attributes in NF (Native Forest), PAS (pasture), GF (Green fertilization), RC (rubber tree cocoa), CAB (Cabruca). The analyzed parameters were SOM (soil organic matter), pH, CEC (cation exchange capacity), V% (percentage saturation by base), m% (percentage saturation by aluminum), P (phosphorus), N (nitrogen), Cu (copper), B (boron), Fe (iron), Mn (manganese), Zn (zinc), Sand, silt and clay. Graphs show the means and standard deviation of the three depths assessed (n=6). Equal letters indicate areas that do not differ between them according to the Kruskal-Wallis test.

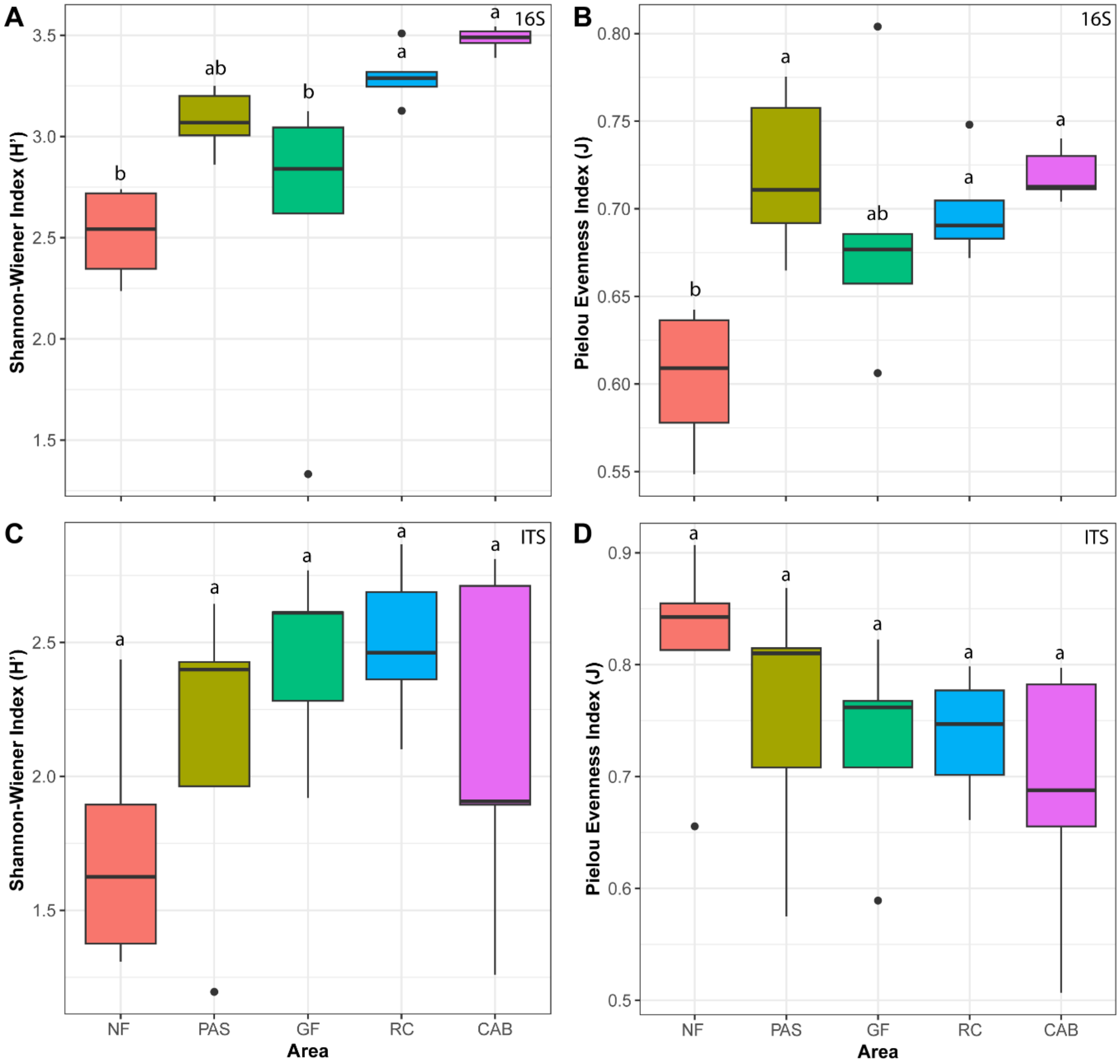

Figure S2. Shannon-Wiener and Pielou Evenness indices obtained by Metabarcoding of bacterial (A and B) and fungal (C and D) samples from NF: native forest, PAS: pasture, GF: green fertilization, RC: rubber-cocoa intercropping; and CAB: Cabruca. Significant differences are marked with different letters.

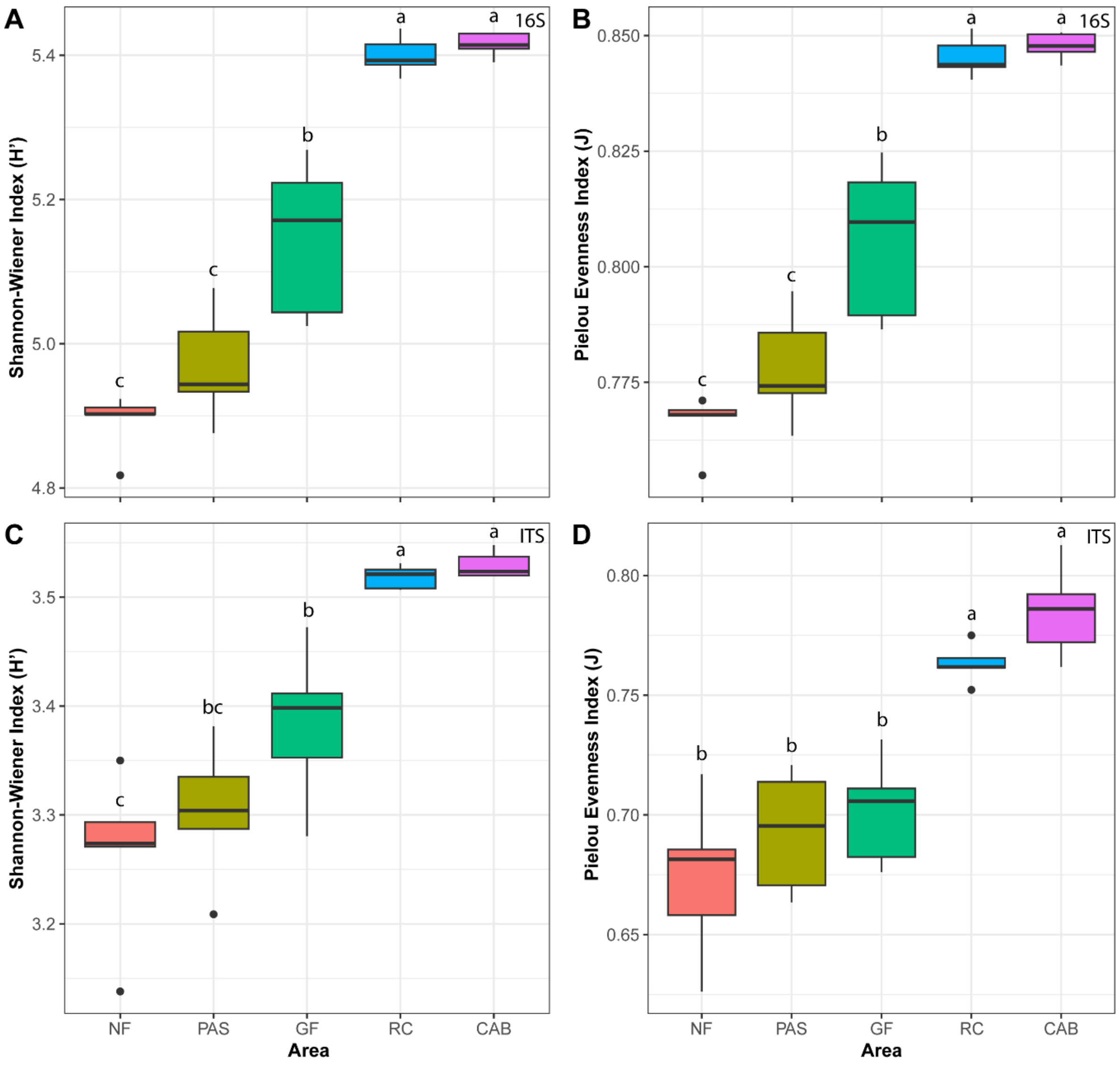

Figure S3. Shannon-Wiener and Pielou Evenness indices obtained by metagenomics on bacterial (A and B) and fungal (C and D) samples obtained from NF: native forest, PAS: pasture, GF: green fertilization, RC: rubber-cocoa intercropping; and CAB: Cabruca. Significant differences are marked with different letters.

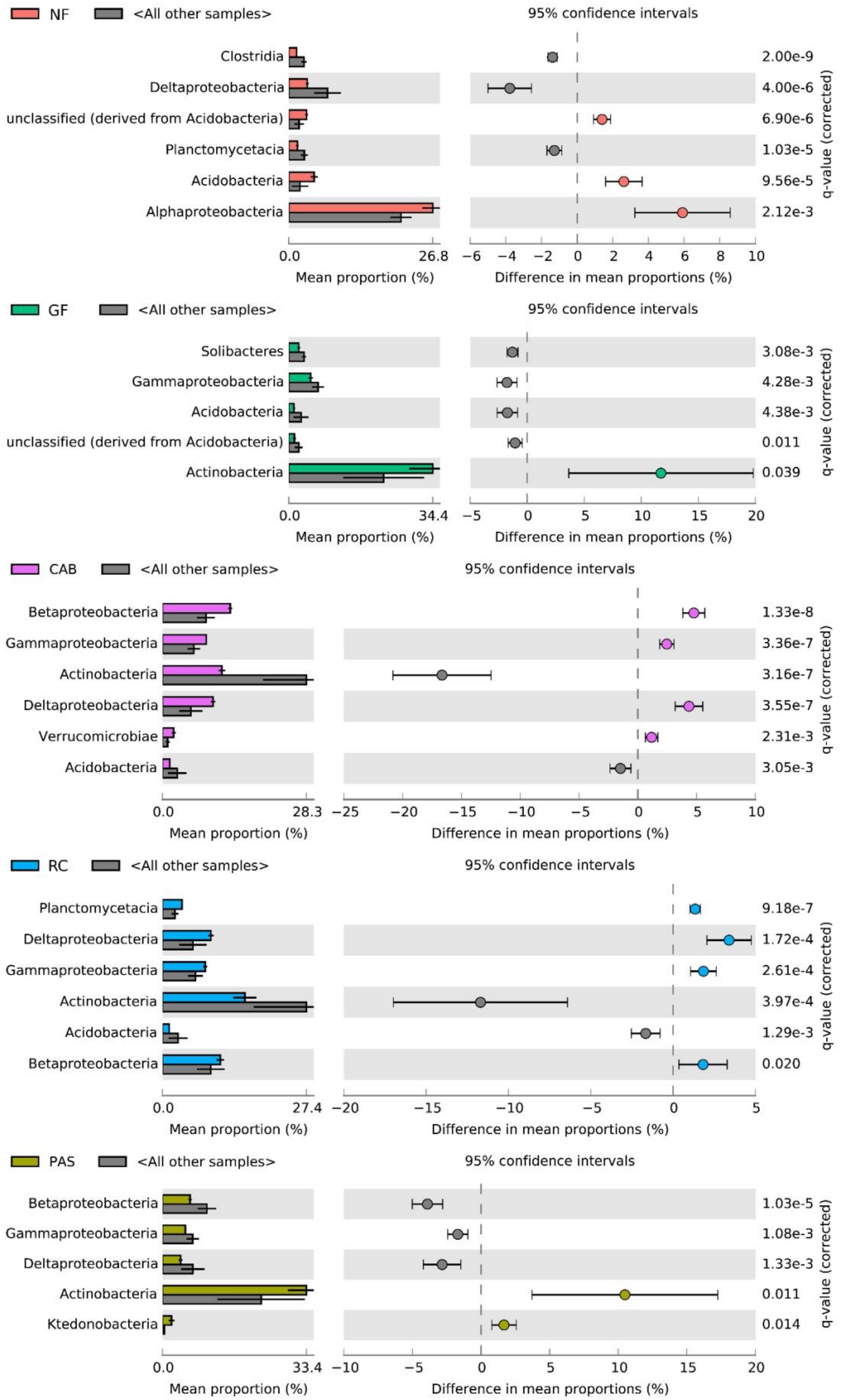

Figure S4. Differential bacterial analysis between the areas NF (native forest), GF (green fertilization), RC (rubber-cocoa intercropping), CAB (Cabruca), and PAS (pasture) at the class level with Benjamini-Hochberg FDR correction ($q < 0.05$).

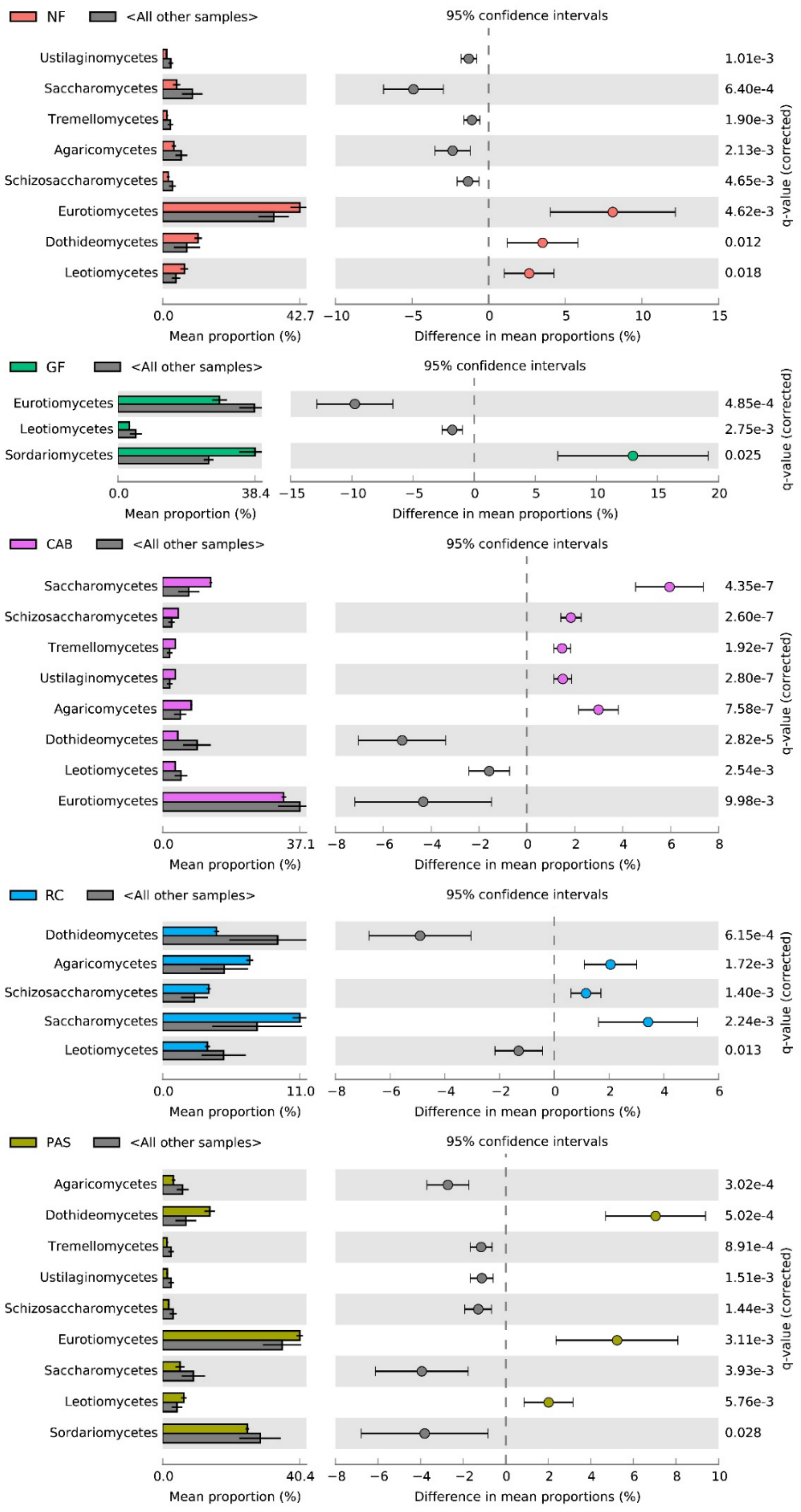

Figure S5. Differential fungal analysis between the areas NF (native forest), GF (green fertilization), RC (rubber-cocoa intercropping), CAB (Cabruca), and PAS (pasture) at the class level with Benjamini-Hochberg FDR correction ($q < 0.05$).

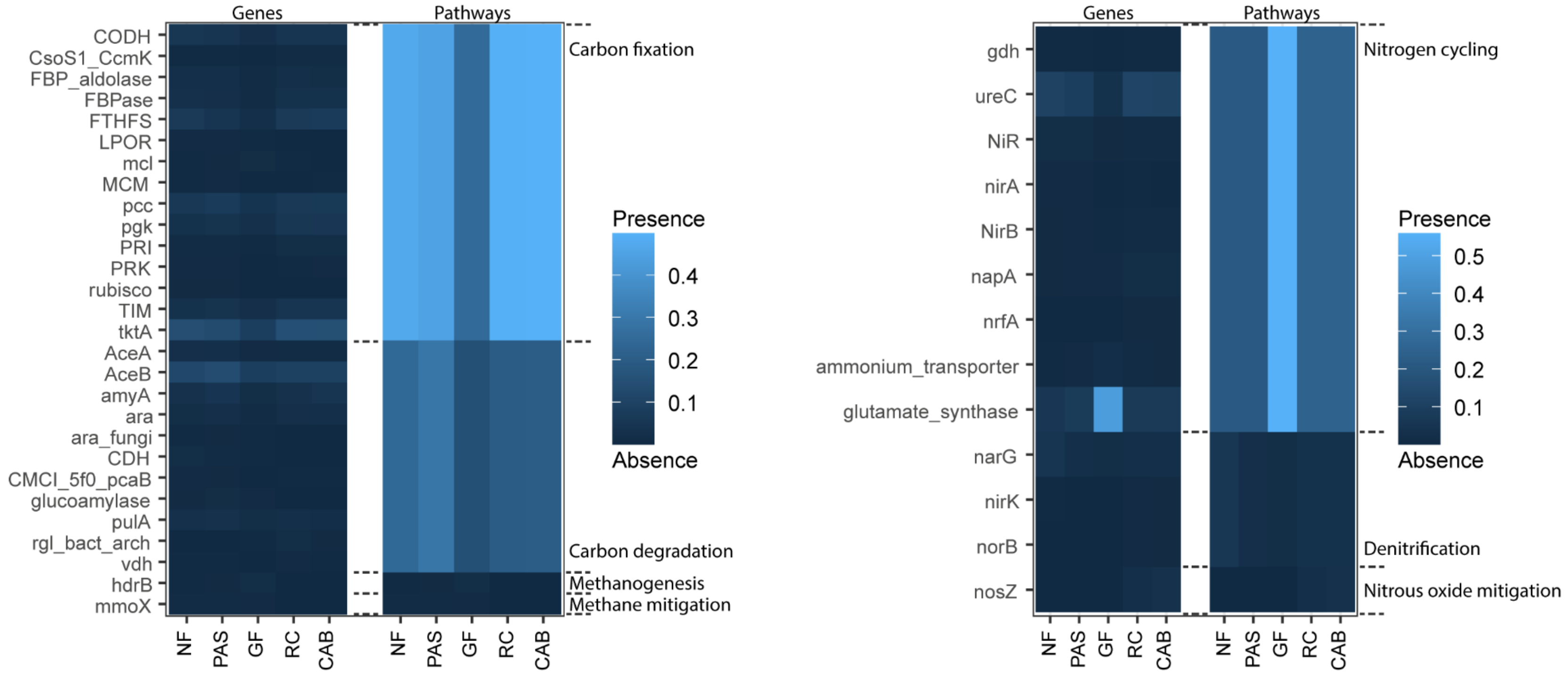

Figure S6. Heatmap showing the abundance of functional genes and metabolic pathways related to carbon (A) and nitrogen (B) transformations in the studied soils. NF: native forest; PAS: pasture; GF: green fertilization; RC: rubber-cocoa intercropping; and CAB: Cabruca.

Table S1. Bacterial 16S rRNA sequences at the genus level obtained from study soil samples: NF: native forest; PAS: pasture; GF: green fertilization; RC: rubber-cocoa intercropping; and CAB: Cabruca.

**Bacterial genera**	**NF ± SE**	**PAS ± SE**	**GF ± SE**	**RC ± SE**	**CAB ± SE**
Acetobacter	0 ± 0	5 ± 1	450 ± 447	0 ± 0	0 ± 0
Acidibacter	433 ± 70	346 ± 48	17 ± 9	42 ± 11	33 ± 2
Acidicaldus	222 ± 53	36 ± 10	15 ± 11	1 ± 1	2 ± 1
Acidothermus	343 ± 61	30 ± 7	3 ± 3	0 ± 0	1 ± 0
ADurb.Bin063-1	2 ± 1	6 ± 1	1 ± 0	3 ± 1	12 ± 3
AKYG587	0 ± 0	0 ± 0	0 ± 0	2 ± 1	7 ± 3
Bacillus	39 ± 15	28 ± 9	52 ± 32	8 ± 2	6 ± 2
Bdellovibrio	1 ± 0	1 ± 1	1 ± 1	1 ± 0	0 ± 0
Bradyrhizobium	180 ± 39	463 ± 64	69 ± 45	67 ± 8	61 ± 10
Bryobacter	212 ± 37	264 ± 65	77 ± 45	11 ± 2	14 ± 4
Burkholderia-Caballeronia-Paraburkholderia	50 ± 10	7 ± 2	0 ± 0	1 ± 0	0 ± 0
Candidatus Koribacter	172 ± 42	19 ± 7	23 ± 17	3 ± 1	32 ± 11
Candidatus Solibacter	512 ± 74	183 ± 35	135 ± 74	147 ± 23	162 ± 15
Candidatus Udaeobacter	11 ± 4	207 ± 109	54 ± 37	80 ± 16	72 ± 7
Candidatus Xiphinematobacter	344 ± 51	141 ± 26	1 ± 1	0 ± 0	0 ± 0
Chryseolinea	0 ± 0	0 ± 0	0 ± 0	1 ± 1	1 ± 0
Crossiella	0 ± 0	8 ± 2	10 ± 4	1 ± 1	2 ± 2
Ellin6067	0 ± 0	0 ± 0	1 ± 1	1 ± 0	3 ± 1
Flavobacterium	0 ± 0	0 ± 0	0 ± 0	18 ± 5	46 ± 11
GOUTA6	0 ± 0	0 ± 0	0 ± 0	1 ± 1	37 ± 4
Lactobacillus	0 ± 0	0 ± 0	172 ± 172	0 ± 0	0 ± 0
Lysinibacillus	12 ± 5	7 ± 3	137 ± 135	2 ± 1	0 ± 0
Methylobacterium	0 ± 0	5 ± 3	6 ± 3	0 ± 0	0 ± 0
MND1	0 ± 0	0 ± 0	3 ± 2	36 ± 6	76 ± 5
Mycobacterium	51 ± 3	12 ± 2	3 ± 2	0 ± 0	1 ± 1
Nitrospira	0 ± 0	0 ± 0	12 ± 5	98 ± 18	202 ± 12
Pajaroellobacter	3 ± 1	1 ± 0	5 ± 2	5 ± 1	7 ± 1
Phaselicystis	0 ± 0	0 ± 0	4 ± 2	8 ± 2	8 ± 2
Pir4 lineage	0 ± 0	0 ± 0	0 ± 0	5 ± 2	2 ± 1
Pseudomonas	0 ± 0	0 ± 0	0 ± 0	8 ± 1	4 ± 2
Ramlibacter	0 ± 0	0 ± 0	1 ± 0	0 ± 0	1 ± 0
RB41	0 ± 0	0 ± 0	0 ± 0	22 ± 4	46 ± 10
SM1A02	0 ± 0	0 ± 0	0 ± 0	4 ± 2	12 ± 4
Solirubrobacter	54 ± 11	54 ± 10	7 ± 4	3 ± 1	2 ± 0
Sphaerotilus	0 ± 0	0 ± 0	0 ± 0	4 ± 1	8 ± 2
Sphingomonas	8 ± 4	302 ± 149	152 ± 73	106 ± 15	107 ± 5
Streptomyces	29 ± 6	63 ± 13	35 ± 18	34 ± 5	17 ± 3
Terrimonas	3 ± 2	18 ± 16	1 ± 0	20 ± 4	8 ± 3
uncultured	3927 ± 30	1613 ± 234	387 ± 222	1022 ± 111	1125 ± 41
uncultured Acidobacteria bacterium	139 ± 29	78 ± 43	5 ± 2	6 ± 1	15 ± 5
uncultured archaeon	0 ± 0	0 ± 0	0 ± 0	0 ± 0	4 ± 1
uncultured bacterium	1397 ± 113	1292 ± 408	134 ± 76	691 ± 91	755 ± 21
uncultured bacterium #0319-6E22	0 ± 0	0 ± 0	0 ± 0	0 ± 0	0 ± 0
uncultured Candidatus Saccharibacteria bacterium	0 ± 0	0 ± 0	1 ± 1	7 ± 2	7 ± 2
Verrucosispora	0 ± 0	0 ± 0	0 ± 0	3 ± 1	3 ± 2
unclassified	759 ± 83	1278 ± 254	328 ± 48	376 ± 54	521 ± 123

Table S2. Fungal 18S rRNA sequences at the genus level obtained from study soil samples: NF: native forest; PAS: pasture; GF: green fertilization; RC: rubber-cocoa intercropping; and CAB: Cabruca.

Fungal genera	NF ± SE	PAS ± SE	GF ± SE	RC ± SE	CAB ± SE
Amanita	0 ± 0	0 ± 0	0 ± 0	2 ± 1	0 ± 0
Agaricus	2 ± 2	0 ± 0	1 ± 1	0 ± 0	2 ± 2
Asterostroma	0 ± 0	2 ± 2	0 ± 0	0 ± 0	0 ± 0
Calvatia	0 ± 0	0 ± 0	0 ± 0	0 ± 0	2 ± 2
Ceratobasidium	0 ± 0	0 ± 0	1 ± 0	0 ± 0	0 ± 0
Claroideoglomus	0 ± 0	0 ± 0	1 ± 1	0 ± 0	0 ± 0
Coprinopsis	0 ± 0	0 ± 0	0 ± 0	0 ± 0	2 ± 2
Cunninghamella	2 ± 1	0 ± 0	0 ± 0	0 ± 0	0 ± 0
Cystobasidium	0 ± 0	0 ± 0	0 ± 0	0 ± 0	7 ± 5
Cystolepiota	0 ± 0	0 ± 0	0 ± 0	2 ± 2	0 ± 0
Entoloma	0 ± 0	0 ± 0	0 ± 0	0 ± 0	2 ± 2
Gymnopilus	2 ± 2	10 ± 5	0 ± 0	0 ± 0	0 ± 0
Hygrocybe	0 ± 0	1 ± 1	0 ± 0	1 ± 1	0 ± 0
Lepiota	0 ± 0	0 ± 0	0 ± 0	1 ± 1	1 ± 1
Leucocoprinus	2 ± 2	0 ± 0	0 ± 0	0 ± 0	0 ± 0
Lycoperdon	0 ± 0	0 ± 0	2 ± 1	11 ± 11	0 ± 0
Lyophyllum	1 ± 1	0 ± 0	0 ± 0	1 ± 1	0 ± 0
Malassezia	0 ± 0	0 ± 0	0 ± 0	1 ± 1	0 ± 0
Mortierella	6 ± 5	1 ± 1	9 ± 7	138 ± 27	115 ± 82
Neopaxillus	0 ± 0	0 ± 0	0 ± 0	0 ± 0	0 ± 0
Ochroconis	0 ± 0	0 ± 0	0 ± 0	4 ± 3	0 ± 0
Pisolithus	0 ± 0	0 ± 0	0 ± 0	2 ± 2	0 ± 0
Pluteus	0 ± 0	0 ± 0	0 ± 0	2 ± 2	0 ± 0
Psathyrella	0 ± 0	0 ± 0	3 ± 2	0 ± 0	0 ± 0
Rhizophlyctis	11 ± 7	2 ± 2	11 ± 6	0 ± 0	17 ± 17
Rhodotorula	0 ± 0	4 ± 4	0 ± 0	0 ± 0	0 ± 0
Saccharomyces	65 ± 17	213 ± 85	96 ± 36	238 ± 72	225 ± 68
Scleroderma	0 ± 0	0 ± 0	0 ± 0	1 ± 1	0 ± 0
Spizellomyces	0 ± 0	0 ± 0	4 ± 2	0 ± 0	0 ± 0
Tomentella	0 ± 0	0 ± 0	0 ± 0	0 ± 0	1 ± 1
Torulaspora	0 ± 0	11 ± 11	0 ± 0	0 ± 0	9 ± 9
Trechispora	1 ± 1	0 ± 0	0 ± 0	2 ± 2	0 ± 0
Tulostoma	11 ± 11	10 ± 10	11 ± 5	0 ± 0	8 ± 8
Verruconis	0 ± 0	0 ± 0	0 ± 0	22 ± 14	0 ± 0
unidentified	6 ± 5	25 ± 9	6 ± 3	68 ± 54	22 ± 15
unclassified	759 ± 83	1278 ± 254	328 ± 48	376 ± 54	521 ± 123
